# *In vitro* higher-order oligomeric assembly of the respiratory syncytial virus M2-1 protein with longer RNAs

**DOI:** 10.1128/jvi.01046-24

**Published:** 2024-07-17

**Authors:** Yunrong Gao, Anirudh Raghavan, Sara Andrea Espinosa Garcia, Bowei Deng, Diego Hurtado de Mendoza, Bo Liang

**Affiliations:** 1 Department of Biochemistry, Emory University School of Medicine, Atlanta, Georgia, USA; University of Kentucky College of Medicine, Lexington, Kentucky, USA

**Keywords:** respiratory syncytial virus (RSV), M2-1 protein, RNA, higher-order oligomer, size exclusion chromatography (SEC), electrophoretic mobility shift assays (EMSA), negative stain electron microscopy (EM), mutagenesis

## Abstract

The respiratory syncytial virus (RSV) M2-1 protein is a transcriptional antitermination factor crucial for efficiently synthesizing multiple full-length viral mRNAs. During RSV infection, M2-1 exists in a complex with mRNA within cytoplasmic compartments called inclusion body-associated granules (IBAGs). Prior studies showed that M2-1 can bind along the entire length of viral mRNAs instead of just gene-end (GE) sequences, suggesting that M2-1 has more sophisticated RNA recognition and binding characteristics. Here, we analyzed the higher oligomeric complexes formed by M2-1 and RNAs *in vitro* using size exclusion chromatography (SEC), electrophoretic mobility shift assays (EMSA), negative stain electron microscopy (EM), and mutagenesis. We observed that the minimal RNA length for such higher oligomeric assembly is about 14 nucleotides for polyadenine sequences, and longer RNAs exhibit distinct RNA-induced binding modality to M2-1, leading to enhanced particle formation frequency and particle homogeneity as the local RNA concentration increases. We showed that particular cysteine residues of the M2-1 cysteine-cysteine-cystine-histidine (CCCH) zinc-binding motif are essential for higher oligomeric assembly. Furthermore, complexes assembled with long polyadenine sequences remain unaffected when co-incubated with ribonucleases or a zinc chelation agent. Our study provided new insights into the higher oligomeric assembly of M2-1 with longer RNA.

**IMPORTANCE:**

Respiratory syncytial virus (RSV) causes significant respiratory infections in infants, the elderly, and immunocompromised individuals. The virus forms specialized compartments to produce genetic material, with the M2-1 protein playing a pivotal role. M2-1 acts as an anti-terminator in viral transcription, ensuring the creation of complete viral mRNA and associating with both viral and cellular mRNA. Our research focuses on understanding M2-1’s function in viral mRNA synthesis by modeling interactions in a controlled environment. This approach is crucial due to the challenges of studying these compartments *in vivo*. Reconstructing the system *in vitro* uncovers structural and biochemical aspects and reveals the potential functions of M2-1 and its homologs in related viruses. Our work may contribute to identifying targets for antiviral inhibitors and advancing RSV infection treatment.

## INTRODUCTION

Respiratory syncytial virus (RSV) is an important human pathogen that causes lower respiratory tract infection in infants, old adults, and immunocompromised patients globally (1–3). RSV belongs to the family *Pneumoviridae* and order *Mononegavirales* (4), which consists of nonsegmented, negative-sense, single-stranded RNA viruses (5). The 15 kilobase (kb) RSV genome contains 10 genes encoding 11 different proteins in the following order: nonstructural protein 1 (NS1), nonstructural protein 2 (NS2), nucleoprotein (N), phosphoprotein (P), matrix protein (M), small hydrophobic protein (SH), glycoprotein (G), fusion protein (F), M2-1 protein, M2-2 protein, and the large (L) protein (6). Each gene is flanked by semi-conserved gene start (GS) and gene end (GE) sequences at the 3′ and 5′ ends (7), respectively. GS and GE act as the initiation and termination signals for synthesizing monocistronic, translatable mRNA sequences. Of these genes, the RSV M2 gene is unique, where it has two open reading frames (ORFs) with a partial overlap of 10 amino acids; the first ORF encodes the M2-1 protein, whereas the second ORF encodes the M2-2 protein (8). Additionally, RSV genes are separated by nonconserved intragenic sequences of various lengths. Just as with other negative-sense single-strand viruses, the transcription of RSV genes is sequential and progresses from the genomic 3′ to the 5′ end (9). This is accomplished by the RNA-dependent RNA Polymerase (RdRP), which in RSV comprises the catalytic large polymerase subunit, L, and its cofactor phosphoprotein, P (10, 11). The RdRP uses a 44-nucleotide (nt) promoter in the 3′ leader region (Le) of the genome to initiate both antigenomic RNA synthesis and mRNA transcription (12), and the presence of GS or GE sequences modulates its activity. Specifically, mRNA synthesis is initiated when the RdRP encounters a GS sequence, with subsequent transcription and capping of the nascent mRNA strand (7, 13). When the RdRP encounters the GE sequence, the mRNA is polyadenylated. Notably, transcriptional attenuation occurs at these intragenic sequences, so genes nearer to the 3' promoter sequence are transcribed more abundantly (14–16). Thus, mRNA synthesis in RSV is thought to follow a gradient pattern. However, ignoring these GS and GE sequences, a full-length antigenome sequence is synthesized during genomic replication.

The RSV M2-1 protein is a basic, RNA-binding protein that is 194 amino acids long and forms a stable tetramer in solution (17). M2-1 consists of three distinct domains: a zinc-binding cysteine-cysteine-cystine-histidine (CCCH) motif at the N terminus (residues 1–31) required for RNA binding, and extension, a central oligomerization domain (residues 32–68) which allows tetramer formation, and a core domain (residues 69–194) that also functions in RNA binding (17–22). In infected cells, M2-1 exists either in a phosphorylated or an unphosphorylated form and interacts with multiple other viral components, such as RNA and P (23). Tetrameric oligomerization is essential for M2-1 activity (17). The M2-1 protein of human metapneumovirus (HMPV) is homologous to the RSV M2-1, and they share commonalities in their folding patterns and tetrameric structural configuration (22, 24). The HMPV M2-1 crystal structure depicted that it is an asymmetric tetramer wherein one protomer is in the open conformation and the other three are in a closed conformation, with the tetramer itself stabilized through interactions between N-terminal zinc-binding domains of adjacent protomers (24). Biophysical studies of the RSV *apo* M2-1 tetramer have demonstrated that it can cycle between a closed and open structural conformation, which could potentially modulate protein function through selective exposure of specific binding sites (25). However, analysis of monomeric configurations of the tetrameric M2-1 in complex with RNA has not yet been fully completed in terms of structure or mechanism, likely due to the highly dynamic nature of the protein itself and the difficulty in chronologically identifying the requisite monomer-monomer or monomer-RNA interactions. The M2-1 protein is neither necessary for the RdRP to initiate mRNA synthesis nor for mRNA capping or polyadenylation (26). Instead, M2-1 functions as a transcription elongation/transcription antitermination factor, and its effect on the synthesis of full-length mRNA is more pronounced when transcribing longer gene lengths—for instance, a prior result has shown that M2-1 increases the level of mRNA transcribed from the 532 nt NS1 gene sequence only by about two to threefold, but increases the level of mRNA transcribed from the 1,780 nt full-length luciferase gene by at least 365-fold (27).

M2-1 has been shown to colocalize within infected cells with terminated viral mRNAs in specialized granules called inclusion body-associated granules (IBAGs) when isolated from viral genomic RNA and the RdRP complex (8, 23, 26, 28, 29). M2-1 recruitment to these inclusion bodies is thought to be mediated via interaction with the P protein, and the M2-1-P complex associates with the RdRP complex (18). M2-1 binds to newly synthesized viral mRNA with dissociation of P, and M2-1 remains associated with viral mRNA even after mRNA release from the IBAG (21, 30). M2-1 is believed to prevent premature transcription termination in these granules by stabilizing the RdRP complex and preventing RNA secondary structure formation. Additionally, M2-1 is thought to exert post-transcriptional control over RSV mRNA expression, possibly through mRNA stabilization, mRNA export to the cytosol, upregulation of mRNA translation, or any combination of these via interactions with gene-end sequences or with the polyadenine tail (18, 21, 22, 30, 31).

In this study, we illustrated that the M2-1:RNA complex is a highly dynamic structure and that its oligomerization potential is highly sensitive to local protein:RNA ratio, colocalized RNA sequence length, and composition. We analyzed the oligomerization capacity of M2-1 and its RNA-binding capacity *in vitro* by manipulating certain key M2-1 residues, utilizing an array of RNA sequences differing in composition and length, and modifying the local M2-1:RNA ratio. This was done through site-specific mutagenesis of M2-1, electrophoretic mobility shift assays (EMSA) of M2-1 with various RNA lengths and sequences, and negative stain electron microscopy (EM) of the M2-1:RNA complex. We defined that the minimal length requirement for higher oligomeric assembly of RNA to M2-1 is at least 14 nucleotides for polyadenine sequences. Additionally, increasing the adenine content in a given RNA sequence resulted in greater particle assembly frequency and greater particle homogeneity. Long RNA binding by M2-1 was RNA-induced and relied on its zinc-binding domain, specifically the M2-1 CCCH motif, and the complex binding functionality was lost when the C7 residue was mutated. Furthermore, our data indicated that the M2-1:RNA complex assembly was responsive to changes in the M2-1-to-RNA molar ratio, wherein increased particle formation and more uniform complexes occurred at higher RNA concentrations. Our findings revealed that M2-1:RNA complexes assembled with polyadenine sequences longer than 14 nucleotides in length remained stable and unperturbed by zinc chelation or RNase A treatment post-RNA-binding. In analyzing an *in vitro* setting resembling *in vivo* IBAGs which form during RSV infection, where M2-1 is compartmentalized with viral mRNA, our study suggests that M2-1 may exhibit a more intricate RNA binding behavior and may engage in post-transcriptional functions that go beyond its conventional role as a transcription antitermination protein.

## RESULTS

### M2-1 binds to RNA sequences in a length-dependent manner

**Fig 1:**
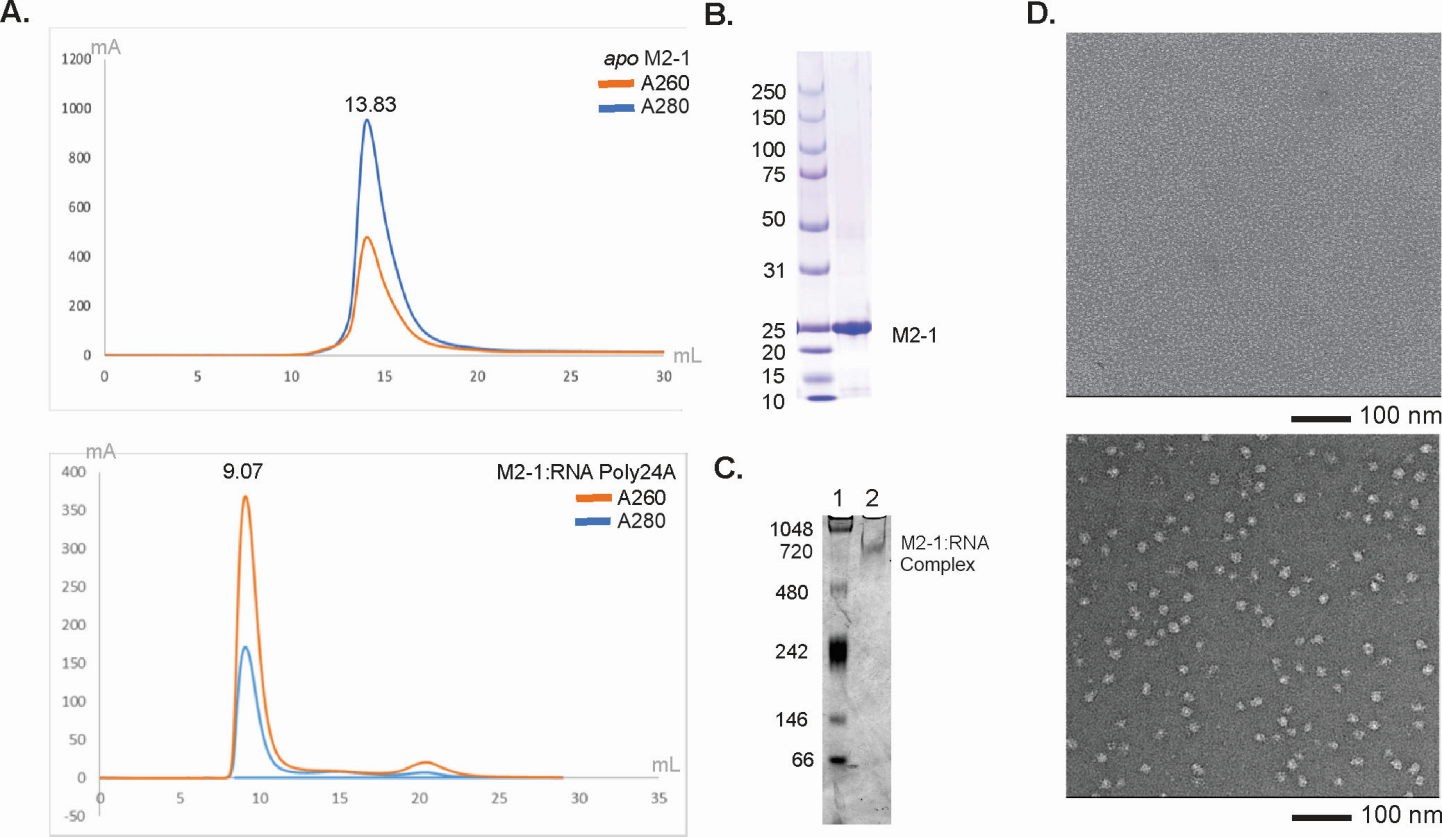
Characterization of M2-1:RNA poly24A Complex through SEC and EM Imaging. (**A**) Gel filtration elution profile of *apo* M2-1 (top) and the M2-1 and poly24A complex (bottom) using Superdex 200. The blue and orange lines represent the 280 and 260 nm absorption, respectively. The elution volume of the *apo* M2-1 is 13.83, and the elution volume of the complex consisting of M2-1 with RNA poly24A is 9.07. (**B**) SDS-PAGE gel run to analyze the eluate of M2-1 with RNA poly24A complex. Additionally, (**C**) examines the relative size of the M2-1:RNA complex. Well 1 is the NativeMark Unstained Protein Standard. Well 2 is the M2-1 and RNA poly24A complex; (**D**) representative negative stain EM image of the *apo* M2-1 and the M2-1 with RNA poly24A complex.

We first isolated the M2-1 with long RNA (poly24A) M2-1:RNA complex with size exclusion chromatography (SEC). Analyzing the complex peaks in the elution profiles, we observed an initial peak emerging at 9.02 mL. This peak corresponds to a complex larger than 670 kDa based on the standard proteins utilized for the gel calibration of the Superdex Increase 10/300 Gl column. The peak with an A260/A280 absorbance ratio of 2.15 signified the presence of the protein:RNA complex. This peak emerged at a lower elution volume than *apo* M2-1, representing a complex significantly larger than *apo* M2-1; the latter is known to be a tetramer with a molecular weight of 96 kDa (Fig. 1A). The purity of the sample was then confirmed using SDS-PAGE gel analysis (Fig. 1B). Additionally, our native gel results, show that the M2-1 and RNA poly24A complex band is around the 720 kDa standard (Fig. 1C).

Negative stain EM images demonstrated that the complexes of M2-1 and RNA were formed and had no signs of aggregation (Fig. 1D). The images also showed that the particle diameter was approximately 20 nm. Though our previous research had shown M2-1 existing in a complex structure with a short RNA sequence (7 nt of the SH gene-end sequence), we did not observe a complex assembly between M2-1 and the short RNA sequence in the gel filtration column that was otherwise observed when M2-1 was incubated with longer RNA.

### M2-1 prefers to form higher oligomer complex RNA sequences containing more adenine at lengths greater than 14 nucleotides

**Fig 2:**
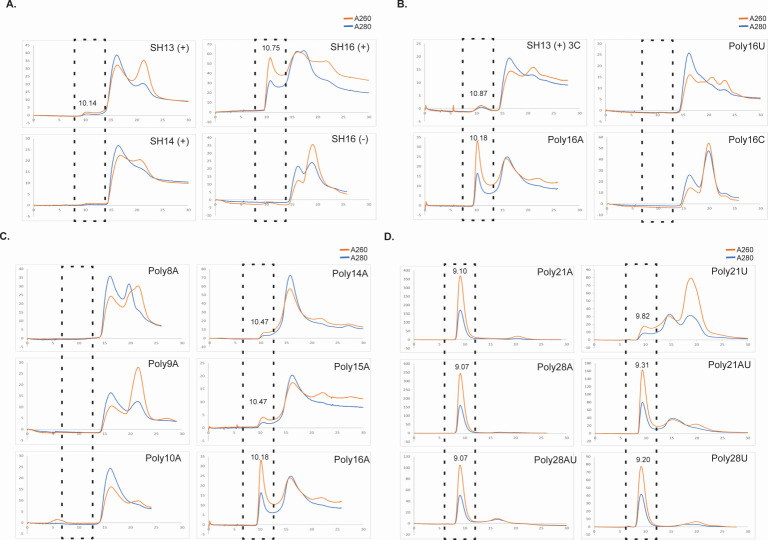
Gel filtration profiles of M2-1 with different RNA oligos. The elution profiles show the major peaks of the M2-1:RNA complex monitored at an absorbance of 280 nm and 260 nm. The region where the complex is expected to elute has been highlighted with a dotted black box. (**A**) Elution profiles of M2-1 and different lengths of RNA SH gene end complexes. (**B**) Elution profiles for M2-1 and the same length of RNA as 16 nt complexes. (**C**) Elution profiles for M2-1:RNA PolyA complexes of differing lengths smaller than Poly24A. (**D**) Elution profiles for M2-1:RNA complexes of polyA, polyU, and polyAU (mixed) sequences.

Prior studies have demonstrated that the transcription antitermination protein M2-1 exhibited a higher binding affinity toward positive-sense strands of gene-end sequences compared to other RNA oligomers. In this study, we aimed to assess the binding activity of various RNA sequences to M2-1 (Table 1). We incubated excess RNA with M2-1 and checked for complex formation based on absorption at A280 and A260. Three peaks were visible, the first peak is M2-1 and RNA complex (A260 > A280), the second peak is M2-1 alone (A280 > A260), and the third peak is free RNA (A260 > A280). We did note some shifting in the elution of the peaks identified, most likely due to the RNA-induced conformational change of *apo* M2-1 or due to the addition of RNA causing interaction of M2-1 with the SEC beads. However, we checked the first peak of the M2-1:RNA complex to test. The positive-sense strand of the 13 nt SH gene-end RNA sequence was tested first to provide a baseline, as prior fluorescence anisotropy binding experiments have illustrated that tetrameric M2-1 has the highest binding affinity to the positive-sense SH gene-end sequence relative to other gene-end sequences (18, 22, 32). Surprisingly, the 13 nt SH gene-end RNA by itself did not show a protein:RNA complex peak on the gel filtration column (Fig. 2A). However, when we extended the 3' end of the gene-end RNA up to 16 nt, the complex peak was observed. In contrast, when we used the same length of 16 nt for the negative-sense strand of the same gene-end RNA sequence, no M2-1 protein and RNA complex peak was detected (Fig. 2A), Indicating complex formation may be dependent on some sequence preference of RNA. Analyzing the sequence composition, we hypothesized that RNA lengths longer than 14 nt and higher percentages of the base adenine (A) in the RNA sequence could be crucial factors contributing to the formation of a stable protein:RNA complex.

**TABLE 1:** List of RNA oligos^*a*^

RNA name	Sequence
SH13(+)	5′-AGUUAAUUAAAAA
SH14(+)	5′-AGUUAAUUAAAAAU
SH16(+)	5′-AGUUAAUUAAAAAUAG
SH16(−)	5′-CUAUUUUUAAUUAACU
SH13(+) 3C	5′-AGUUAAUUAAAAACCC
Poly16A	5′-AAAAAAAAAAAAAAAA
Poly16U	5′-UUUUUUUUUUUUUUUU
Poly16C	5′-CCCCCCCCCCCCCCCC
Poly8A	5′-AAAAAAAA
Poly9A	5′-AAAAAAAAA
Poly10A	5′-AAAAAAAAAA
Poly14A	5′-AAAAAAAAAAAAAA
Poly15A	5′-AAAAAAAAAAAAAAA
Poly16A	5′-AAAAAAAAAAAAAAAA
Poly21A	5′-AAAAAAAAAAAAAAAAAAAAAA
Poly21U	5′-UUUUUUUUUUUUUUUUUUUUU
Poly21AU	5′-WWWWWWWWWWWWWWWWWWWWW (mixed bases W: A, U)
Poly28A	5′-AAAAAAAAAAAAAAAAAAAAAAAAAAAAA
Poly28U	5′-UUUUUUUUUUUUUUUUUUUUUUUUUUUU
Poly28AU	5′-WWWWWWWWWWWWWWWWWWWWW WWWWWWW (mixed bases W: A, U)

^*a*^ The sequences of different oligos used to generate the gel filtration profiles in Fig. 2.

In subsequent experiments, we maintained the RNA length at 16 nt and appended three cytosine bases at the end of the positive-sense strand of the SH gene-end sequence to compare the resultant binding activity with that of poly16A, poly16U, and poly16C RNA sequences with M2-1 (Fig. 2B). The results revealed that only the RNA poly16A could effectively bind with M2-1, resulting in the formation of a protein:RNA complex peak of notable absorbance observed on the gel filtration chromatography profile. This indicated that, despite having the same length of 16 nt, M2-1 exhibited a clear preference for binding to the polyA sequence. The lack of binding with poly16U and poly16C suggests a specific recognition of the adenine-rich sequence by M2-1, emphasizing the significance of the base composition of an RNA sequence in determining the binding affinity between M2-1 and RNA. We did not test polyguanine sequences because no signal was detected in the gel filtration analysis for either the free RNA or the M2-1:RNA complex; this is likely due to the capability of polyG sequences to form ordered and sophisticated secondary structures, namely G-tetrads and G-quadruplexes (33, 34).

### Exceeding an RNA length threshold reduces the RNA nucleotide preference of M2-1

Since M2-1 shows a preference for binding to adenine bases, we further investigated this preference to extract the possibility of an RNA length dependency and test binding patterns when using RNA sequences composed of one specific nucleotide. We began with utilizing polyA RNA sequences for M2-1:RNA complex assembly, and we conducted gel filtration chromatography experiments using RNA sequences ranging from poly8A to poly16A. The results showed that complex peaks appeared when the RNA length reached poly14A, indicating that a minimum length of poly14A is required for stable complex formation with M2-1 (Fig. 2C). Interestingly, as we increased the RNA length beyond poly14A to 21A and 28A and even substituted polyU sequences for polyA sequences at these lengths, stable M2-1:RNA complex peaks were still observed on the gel filtration chromatogram (Fig. 2D). Combining the data from both testing arrays, we found that while M2-1 indeed preferred binding to adenine bases, the crucial factor for binding shifted from base type to RNA length when the RNA sequence length exceeded a certain threshold. This suggests that both base preference and RNA length play a significant role in determining the RNA-binding capacity of M2-1.

### RNA recognition of M2-1 depends on RNA length and the M2-1:RNA ratio

**Fig 3:**
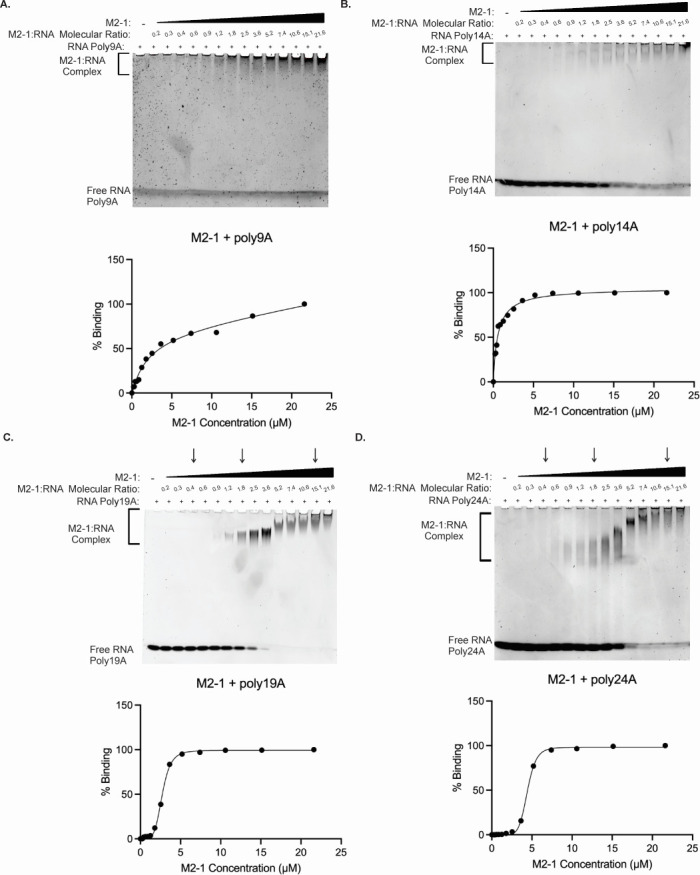
EMSA experiments. (**A–D**) Presents the results of EMSA experiments, investigating the binding interactions between protein M2-1 and different RNA oligos (Poly24A, Poly19A, Poly14A, and Poly9A) at with gradient changed molecular ratio of M2-1 with RNA. Each panel displays the EMSA results for a specific RNA oligo. The molecular ratio between M2-1 and RNA is indicated above the corresponding lanes, and the final RNA concentration used in the binding reactions is 0.5 µM. Corresponding binding curves with the highest goodness of fit are shown below the gel images. (**B–D**) The presented fitted curves were determined from one-site specific binding with Hill slope saturation binding analyses. (**A**) The presented fitted curve was determined from the one-site total and nonspecific saturation binding analysis.

The RNA binding characteristics of M2-1 were evaluated using RNA gel shift assays with various polyA RNA sequences of different lengths: poly9A, poly14A, poly19A, and poly24A (Fig. 3A through D). PolyA sequences were utilized due to the notable binding affinity of M2-1 for such sequences, consistent with our prior results. In each assay, the RNA concentration was fixed at 0.5 µM, while the concentration of the M2-1 protein was incrementally increased by 1.4-fold. The gel shift assay results revealed distinct patterns for the poly24A, poly19A, and poly14A RNA sequences (Fig. 3B through D). As the concentration of M2-1 increased in each assay, the bands corresponding to free RNA oligos showed noticeable reductions in intensity, while the bands representing the M2-1 with RNA complex exhibited increased intensity. A cooperative binding characteristic was observed in the gel shift assays of M2-1 with poly19A and poly24A with representative sigmoidal binding curves (Fig. 3C and D). Conducting specific binding with Hill slope analyses for the gel images, the R² values were 1.00 for both the M2-1 with poly19A and M2-1 with poly24A curves. The Hill coefficient associated with M2-1 binding with poly19A was 5.17 with a 95% confidence interval of (4.53, 6.00), and the Hill coefficient associated with M2-1 binding with poly24A was 8.10 with a 95% confidence interval of (7.39, 8.87); our curve fitting demonstrated that positive cooperativity may be implicated in the binding of M2-1 to these two RNA lengths. In parallel, we also set up a sample of M2-1 with a short poly9A RNA sequence as the control, wherein the significant cooperative binding characteristic observed in the regular gel shift assays could not be seen (Fig. 3A). For our control, the protein and RNA complex bands remained the same size and increased in intensity uniformly with higher protein concentrations, and the corresponding binding curve was hyperbolic in shape (Fig. 3A). The total and nonspecific binding analysis provided a better fitting curve than the specific binding with Hill slope analysis, although the difference was marginal (R² values of 0.99 and 0.98, respectively), and the Hill coefficient obtained for M2-1 binding with poly9A was 0.8282 with a 95% confidence interval of (0.57, 1.13). Interestingly, gel image analysis for M2-1 binding with poly14A did not suggest definitive cooperative binding nor the lack thereof (Fig. 3B). Though the specific binding with Hill slope analysis produced a curve with the strongest fit (R² =0.99), the Hill coefficient associated with the binding interaction was 0.95 with a 95% confidence interval of (0.74, 1.19). The K_d_ values and their 95% confidence intervals associated with the M2-1 binding to the aforementioned polyA sequences were 1.944 µM and (1.05, 3.80), 0.58 µM and (0.48, 0.75), 2.72 µM and (2.65, 2.80), and 4.44 µM and (4.36, 4.51) for binding poly9A, poly14A, poly19A, and poly24A sequences, respectively. These values are estimates generated through hyperbolic/quadratic curve-fitting to a nonspecific binding model using the Prism software and reported to two decimal places, consistent with kinetic measurement techniques detailed in prior studies (35).

Overall, our findings suggest that though the M2-1 protein exhibits preferential binding to polyA sequences, a higher-order oligomeric assembly with cooperative binding characteristics is demonstrated when interacting with longer RNA sequences (poly19A and poly24A). This interaction is further modulated by encapsulated RNA length and protein:RNA ratio during co-incubation, highlighting the complexity and specificity of the M2-1 and RNA interactions.

### The cysteine-7 residue of the M2-1 zinc-binding motif is crucial for the higher-order oligomeric binding of RNA

**Fig 4:**
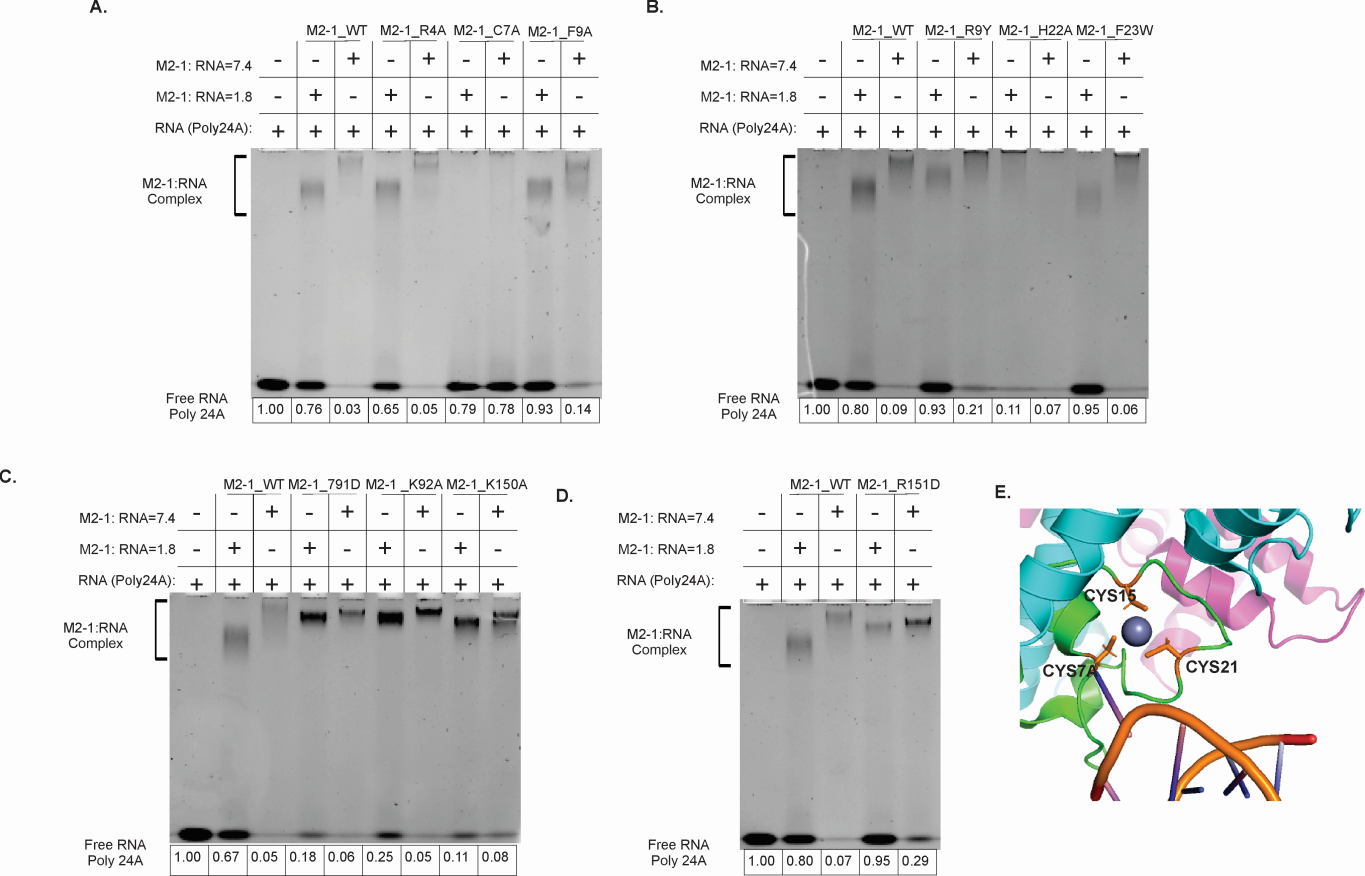
EMSA experiments of M2-1 mutant with RNA. The results of EMSA experiments focus on the binding reactions of various M2-1 mutants RNA poly24A. These experiments were conducted using two specific protein and RNA ratios: 7.4 and 1.8. In figures A–D, the first three wells function as references: well 1 is a negative control, and wells 2 and 3 are positive controls. (**A**) EMSA results for M2-1 mutants R4A, C7A, and F9A. (**B**) EMSA results for M2-1 mutants F9Y, H22A, and F23W. (**C**) EMSA results for M2-1 mutants T91D, K92A, and K150A. (**D**) EMSA results for M2-1 mutant R151D. (**E**) Specific cysteine residues of the zinc-binding motif of RSV M2-1 are highlighted (C7, C15, and C21).

Previous studies investigating the interaction of M2-1 with gene end RNA sequences (<15 nts) identified conserved regions located in the zinc-binding domain and the core domain crucial for RNA binding. We employed two specific M2-1:RNA ratios (7.4 and 1.8), which were both able to potentially elicit cooperative binding characteristics, to examine the interaction between mutated M2-1 and long RNA (Fig. 4E). After preparing and purifying the mutant M2-1 proteins, we conducted RNA gel shift assays to assess the differences in their binding activity (Fig. 4A through D). Interestingly, the RNA gel shift results revealed the simultaneous presence of both the protein:RNA complex and free RNA at the lower ratio (M2-1:RNA = 1.8). When comparing the mutants with the wild-type M2-1:RNA complex, we found that the mutants H22A, T91D, K92A, and K150A exhibited reduced free RNA at a lower ratio. However, the most prominent difference was observed in the mutants of M2-1 C7A (Fig. 4A), which displayed no bands signifying complex formation and the free RNA bands of equal intensity at both high and low ratios. These findings suggest that the cysteine residue at position 7 is a key determinant for the RNA binding character of M2-1 to form a higher oligomer complex, regardless of the ratio between M2-1 and RNA (Fig. 4E). The C7A mutant eluded at the same position as the *apo* M2-1 on SEC, with a wider tailing peak. This suggests the tetrameric formation of M2-1 is still possible but may have reduced stability. The peak fractions (the same as *apo* M2-1) were collected for this analysis.

The identification of this crucial residue sheds light on the molecular basis of the interactions between M2-1 and RNA and provides valuable insights into the mechanisms underlying the role of M2-1 in RNA regulation.

### 2D classification of the M2-1:RNA complex particles reveals distinct morphological trends

**Fig 5:**
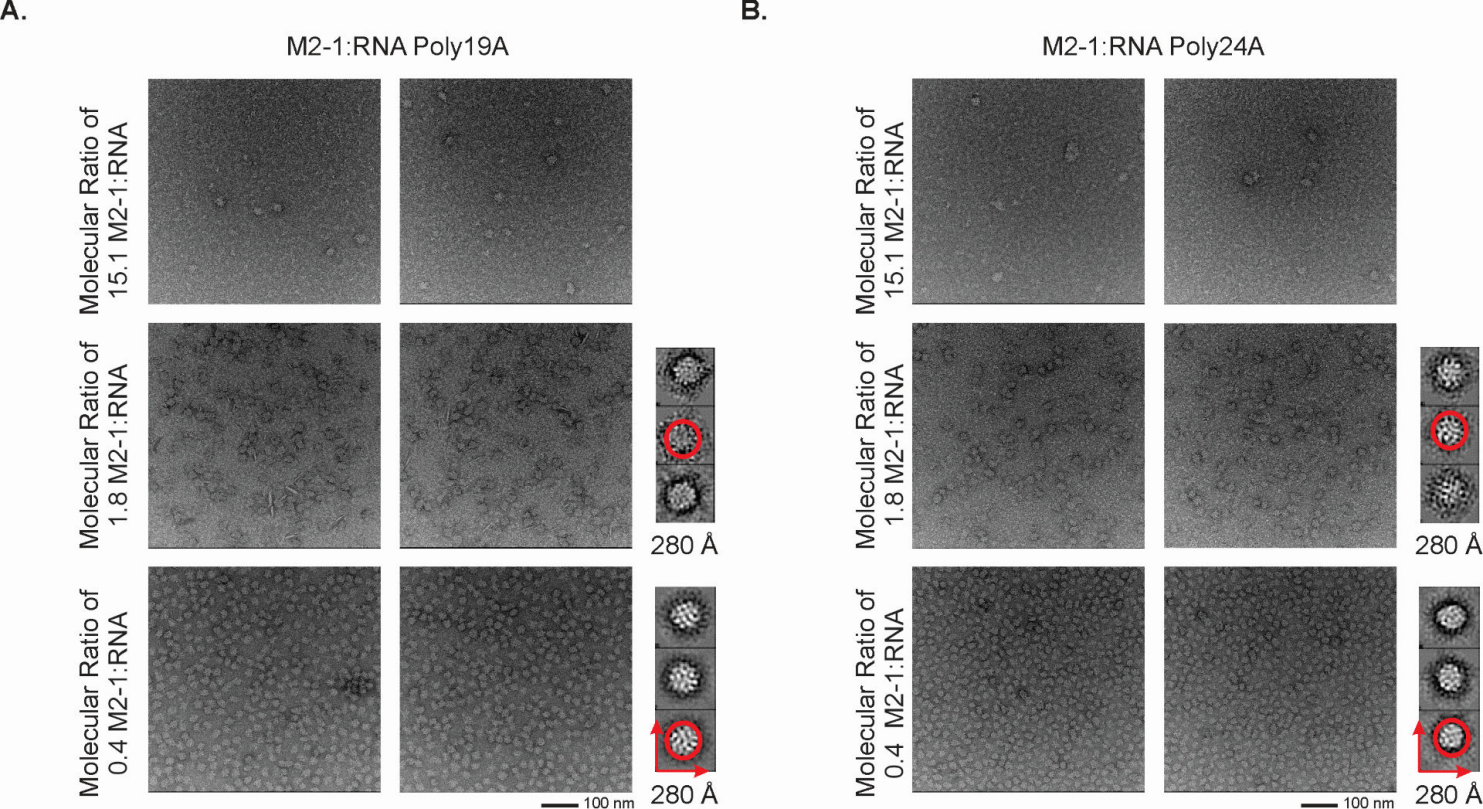
Characterization of M2-1:RNA complex by EM imaging and 2D classification. Representative field of M2-1 with RNA poly 19A (**A**) and poly 24A (**B**) complex from a micrograph acquired at 92,000 nominal magnifications. The concentration of protein M2-1 was fixed at 10.8 µM, and the protein and RNA complex was made based on the following M2-1:RNA ratios: 15.1, 1.8, and 0.4, respectively. 2D class-averaging images for the M2-1 with RNA poly24A and M2-1 with RNA poly19A complexes are included beside the negative stain images wherein substantial particle formation was observed, as was the case with the 1.8 and 0.4 M2-1:RNA ratios for both the poly19A and poly24A RNA sequences.

The gel shift results vividly showcase the formation of distinct M2-1:RNA complexes, varying in size with different RNA concentrations, which were modified through a gradient series of protein-to-RNA ratios (Fig. 5). Notably, as the ratio of M2-1 to RNA increased, the resulting complexes exhibited an incremental increase in size. Maintaining consistent protein concentration, we fixed the ratio based on the gel shift assays (Fig. 3), allowing the RNA concentration to adjust accordingly. Three specific M2-1:RNA ratios (15.1, 1.8, 0.4) were selected for comparison. We uncovered a range of M2-1 complex particle sizes through negative stain EM imaging at the 15.1 M2-1:RNA ratio. Notably, *apo* M2-1, with a molecular weight of approximately 96 kD, did not exhibit a distinctive particle morphology under the same 93K magnification. Of particular interest, the M2-1:RNA complexes showcased greater homogeneity at the 15.1 and 1.8 M2-1:RNA ratios. Moreover, these complexes were slightly larger than those observed at the 0.4 ratio. Using the 2D classification approach to investigate the M2-1:RNA particles, we determined the complex diameter to be approximately 140 Å, while *apo* M2-1 had a diameter of less than 10 Å. This discrepancy indicated the presence of multiple M2-1 units within the M2-1:RNA complexes. Comparing the three distinct M2-1:RNA ratios, we ascertained that elevated RNA levels resulted in greater particle formation propensity and greater homogeneity of the resultant particles. Specifically in the EM images capturing M2-1:RNA particles when co-incubated with a 0.4 M2-1:RNA ratio, the significant variance in particle size and the presence of particle aggregates illustrated that the local concentration of RNA may induce cooperative binding characteristics in M2-1 (Fig. 5A and B). Complex assembly with alterations in M2-1:RNA ratios unveiled a nuanced landscape, wherein their relative concentrations modulated complex size, uniformity, and compactness. We assume that one M2-1 tetramer has the potential to bind to one (minimal occupancy), two, three, or more RNA oligos. We can make estimations based on the molecular weight of one M2-1 tetramer and one oligo of poly24A RNA, which are approximately 96 kDa and 7.8 kDa, respectively. Therefore, each M2-1 tetramer binds to one RNA oligo in the minimal occupancy, resulting in a complex containing roughly 6 M2-1 tetramers and 6 RNA oligos. Alternatively, if each M2-1 tetramer binds to 4 oligos of RNA poly24A, the complex could consist of 5 M2-1 tetramers and 20 RNA oligos. The exact binding mode and composition will need further analysis.

### Bound M2-1:RNA complexes remain stable in the presence of ribonucleases and zinc chelation

**Fig 6:**
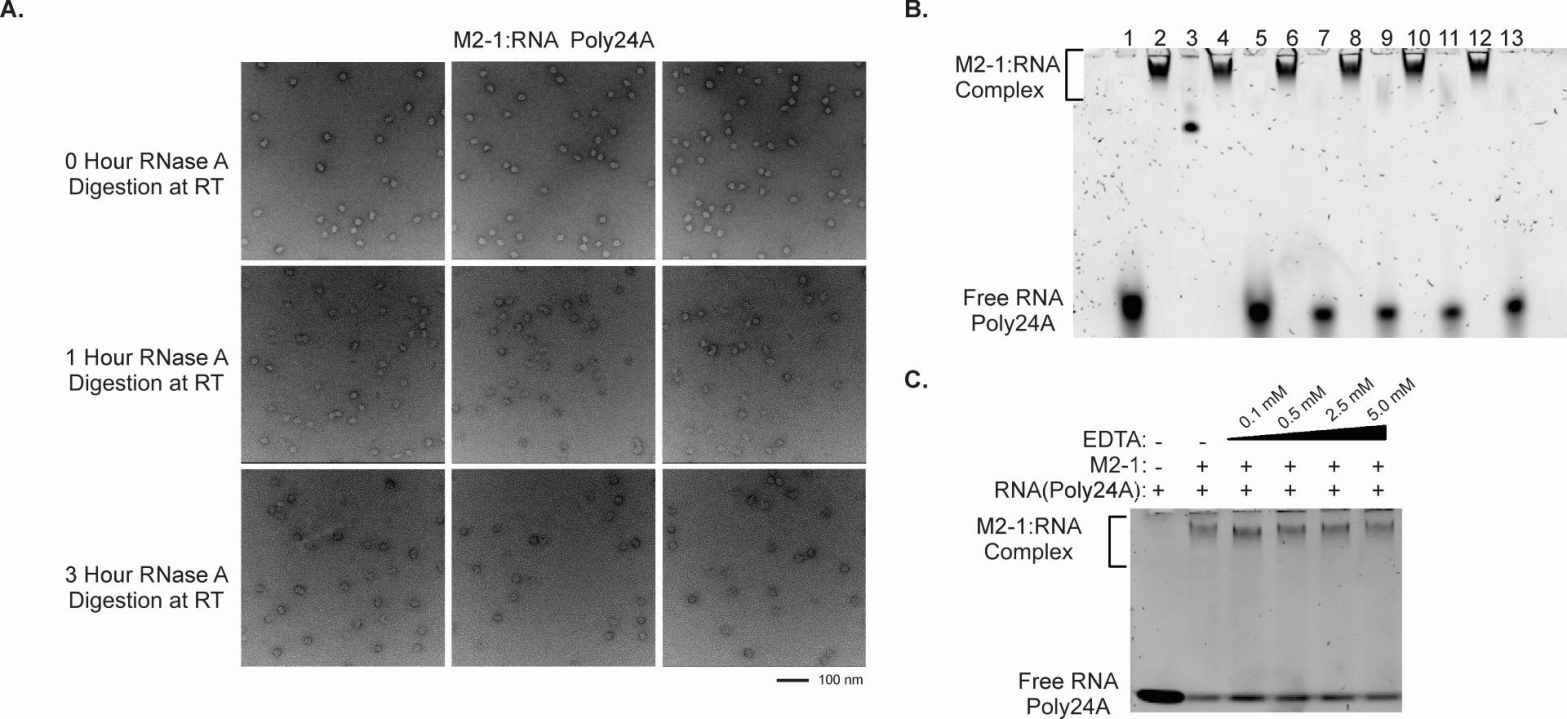
The stability analysis of the M2-1:RNA poly24A complex. (**A**) RNase A digestion was employed to investigate its stability, with negative stain EM imaging performed at 0, 1, and 3 hours at room temperature (RT). (**B**) examines the RNA digestion for up to 5 hours. The wells labeled 1–13 are further described as follows: 1. M2-1 and RNA poly24A complex; 2. RNA poly24A; 3. NativeMark Unstained Protein Standard; 4. M2-1 and RNA poly24A complex after RNaseA digestion at RT for 1 hour; 5. RNA extraction from the M2-1 and RNA poly24A complex from well 4; 6. M2-1 and RNA poly24A complex after RNaseA digestion at RT for 2 hours; 7. RNA extraction from the M2-1 and RNA poly24A complex from well 6; 8. M2-1 and RNA poly24A complex after RNaseA digestion at RT for 3 hours; 9. RNA extraction from the M2-1 and RNA poly24A complex from well 8; 10. M2-1 and RNA poly24A complex after RNaseA digestion at RT for 4 hours; 11. RNA extraction from the M2-1 and RNA poly24A complex from well 10; 12. M2-1 and RNA poly24A complex after RNaseA digestion at RT for 5 hours; 13. RNA extraction from the M2-1 and RNA poly24A complex from well 12. (**C**) Examine the complex's stability under varying conditions through EDTA chelation with increasing concentrations of EDTA. The concentration of EDTA is labeled above.

To investigate the structural integrity of the M2-1:RNA complex, we performed RNase A digestion assays. Negative stain EM images were used to assess any changes in the complex particles upon RNA digestion. The digestion assays were conducted at room temperature for 1 hour to 5 hours, and the samples were separately imaged using negative stain EM (Fig. 6A). Surprisingly, the RNase A digestion did not cause any significant changes in the number of particles, and there were no noticeable differences in the shape or size of the particles, and RNA still can be isolated from the complex after the RNase A digestion. This suggests that the RNase A digestion did not have a significant impact on the structural integrity of the M2-1:RNA complex, and in the native gel result (Fig. 6B), the M2-1 and poly24A complex is stable after the RNase A digestion for up to 5 hours. In addition, we employed a gradient concentration of EDTA for the chelation of zinc from the M2-1 protein (Fig. 6C). The binding activity of M2-1 to RNA was assessed using RNA gel shift assays. Interestingly, as the concentration of EDTA gradually increased, the bands representing the M2-1:RNA complex showed no changes. This indicated that the binding activity of M2-1 to RNA remained unaffected by the zinc chelation from the protein.

## DISCUSSION

The M2-1 protein is the RSV transcription antitermination factor, extensively characterized as the gene-end sequence binding protein (28). M2-1 is required for RSV transcription but is not necessary for RNA replication (26). The results of M2-1 immunoprecipitation assays within infected cells also remain consistent with the notion that M2-1 is predominately bound to RSV mRNA (19). M2-1 is a tetramer with three distinct domains. Near the N-terminus is the CCCH zinc-binding motif, which always plays a role in RNA binding and elongation. The central domain is the oligomerization domain, which plays a key role in M2-1 tetramerization. The core domain also has RNA binding properties. Specifically, two protomers associate with the RNA strand through their zinc-binding motifs, and two protomers associate with the phosphate backbone of the RNA strand through their core domains, giving rise to both RNA-independent and RNA-dependent interactions (20). Whether these M2-1 interactions with RNA are maintained with longer lengths of RNA or with different RNA compositions is yet to be determined. A prior study analyzed RNA recognition of M2-1 and reported that two 20-mer RNA molecules bound to the M2-1 tetramer positively cooperatively within contiguous RNA-binding sites (36). Overall, previous results have indicated that the functions of M2-1 range beyond its traditional roles as a transcriptional processivity and antitermination factor.

The exact reasons for the formation of cytoplasmic IBAGs during RSV infection *in vivo* are yet to be fully characterized. Still, it is known that M2-1 and viral mRNA are compartmentalized within these granules with the exclusion of genomic RNA, the nucleocapsid proteins N and P, as well as the RNA polymerase L protein. Additionally, it has been illustrated previously that RNAs of a sufficient length can bind cooperatively with M2-1 (36), and we speculate that this cooperative binding tendency may have a vital involvement in the formation of IBAGs and successful RSV replication. To probe the possibility that M2-1 may be implicated in post-transcriptional RNA processing, we aimed to elucidate specific M2-1 and RNA binding interactions by varying a range of RNA sequence parameters and mutating selected M2-1 residues. Additionally, we assessed complex assembly with SEC and particle morphologies with EMSA and through negative stain EM imaging followed by 2D classification of the micrographs.

The isolation and characterization of the M2-1 and long RNA complex through gel filtration column methods signify successful complex formation. Specifically, the observed presence of an absorbance peak at a point in the chromatogram signifying higher molecular size relative to *apo* M2-1 suggests the formation of a protein:RNA complex. Our results indicate that short RNA and long RNA to M2-1 binding modalities differ. Still, an interesting result was that we could not observe an absorbance peak representing the M2-1:RNA complex when incubating M2-1 with a 7-nt truncation of the SH gene-end sequence, a structure we could characterize previously (20). However, this may also be attributed to the significantly lower binding affinity that M2-1 has for shorter relative to longer RNA sequences, such that the peak is unremarkable on the gel filtration profile, though the complex itself forms transiently. Calculating the specific M2-1 binding constants with a range of short and long RNA sequences is essential to investigate this further. Negative stain EM imaging aids in visually confirming homogeneity without aggregation. We identify that the M2-1 protein forms stable complexes with long polyadenine RNA sequences and depicts a differential binding behavior between positive-sense strand versus negative-sense strand RNA sequences, which we illustrate using gene-end RNA oligos. Positive-strand sequence RNAs were not solely sufficient in constructing the M2-1:RNA complex, but extending the RNA sequence to 16 nt resulted in complex formation. It is also noted that the complex formation depicted in our SEC experiments could have varied results due to variations in the complex’s elution profile. This is expected, due to the heterogeneous nature of the complex. The binding between M2-1 and RNA results in a complex that may vary in the way that it forms, specifically having different ratios of M2-1 to RNA molecules, which could lead to a great range of end products. The absence of complex formation with the same length of negative-strand RNA further highlights the distinct binding mechanisms between different RNA lengths. Specifically, how the longer length of RNA gives way to complex formation not previously seen with short RNA. This could be an important factor, in the role of M2-1 after termination when M2-1 binds to the newly transcribed RNA, may specifically at the poly(A) tail.

The preference of M2-1 for binding to adenine-rich sequences is evident from the successful formation of a protein-RNA complex with poly16A. The lack of binding with poly16U and poly16C underscores the specific affinity of adenine-rich sequences. The preference for adenine-rich regions may be related to the downstream binding of M2-1 to the capped, polyadenylated mRNA, potentially giving some insights into this phenomenon. An explanation is that the polyA tail plays a role in the movement of M2-1 into granules within the cell. Exploring the preferential binding of M2-1 and its effects is a future direction of our work. The significance of the preferred base composition is further supported by the requirement for a minimum length of poly14A for stable complex formation, demonstrating the interplay between base preference and RNA length. The role of M2-1 in RSV infection is thought to be more complex than simply functioning as an anti-terminator. To further understand how specific preferences of M2-1 binding to RNA affect the function of M2-1, further studies must be performed. During RSV infection *in vivo*, M2-1 is clustered with viral mRNA into sub-compartments called inclusion bodies; possessing a higher affinity for adenine would aid in binding to RNAs that have been post-transcriptionally modified, and an affinity for positive sense as opposed to negative sense illustrates the ability of M2-1 to potentially distinguish between genomic RNA and mRNA. Our findings lend credence to past results displaying that M2-1 clusters with viral mRNA rather than genomic RNA in inclusion bodies. Together, these findings emphasize the potential for a greater binding affinity of M2-1 to RNA to sequences at least 13 nt in length. Furthermore, when the length is equal, M2-1 prefers binding to RNA sequences of primary adenine.

**Fig 7:**
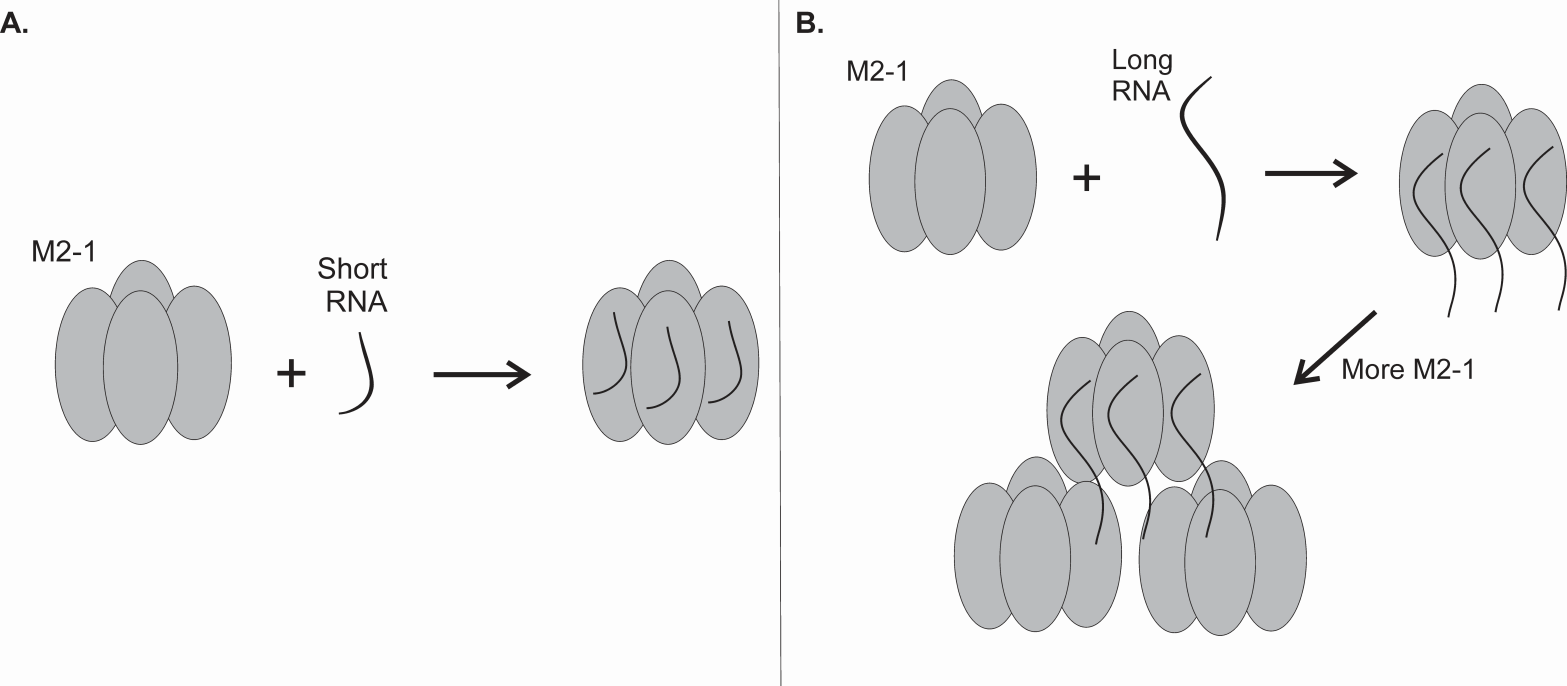
Potential binding modality of M2-1 with short RNA versus longer RNA. The diagram illustrates the potential binding modalities of M2-1 with short and long RNA. (A) When M2-1 binds with short RNA, the entire length of the RNA sequence is bound by a single M2-1, leading to a binding stoichiometry of 1:1. (B) A longer RNA sequence allows for more space and thus allows the binding of multiple M2-1 proteins to a single RNA strand.

The higher oligomeric assembly of M2-1 with longer RNA sequences, such as poly19A and poly24A, indicates a more intricate binding mechanism, which is modeled in Fig. 7. This suggests that the interactions of M2-1 with RNA may involve sequential conformational changes or multivalent interactions that are RNA length-dependent. While the binding modalities of poly19A and poly24A with M2-1 appear to be characterized by positive cooperativity, the binding modality of poly9A does not show any significant positive cooperativity. However, M2-1 with poly14A seems to be the length corresponding to the cusp of the transition from one mode of RNA binding to the RNA sequence eliciting positive cooperativity. This roughly aligns with prior results, wherein it was noted that 13 nt was the threshold beyond which RNAs bind cooperatively to M2-1 (36). The K_d_ values we calculated were significantly divergent from previously published results regarding tetrameric M2-1 binding to similar lengths of polyadenylated RNAs: prior data regarding the dissociation constant value for the wild-type M2-1 tetramer binding to poly13A was determined as 19.1 ± 3.37 nM (22), and the K_d_ for the binding of a 20-mer RNA corresponding to the SH protein GE sequence was determined as 20 nM (37). These concentrations were not in the range of the concentrations of M2-1 that were used in our trials, and this may be a result of the variance in M2-1:RNA binding stoichiometry that may be RNA-length dependent, or due to preferential protein-protein or protein-RNA interactions such that there is a reduction in RNA binding capability, perhaps by sequestration or occupation of the RNA binding cleft. Additionally, longer RNA lengths and sequence compositions may bind in an even more complex and variable manner than previously elucidated. Reiterating the point about binding stoichiometry, the total percentage of bound RNA may vary drastically for the different lengths of RNA we tested as the M2-1 concentration was altered in a uniform gradient for all trials. Per our results, M2-1 having a greater preference for binding to poly14A as opposed to poly9A is expected, but the increase in K_d_ values for poly19A and poly24A relative to poly9A and poly14A is quite counterintuitive. This may suggest that specific RNA lengths may be preferred to the extent that the generally positive correlation between RNA length and M2-1 may be obfuscated. Conversely, there may be very fine M2-1:RNA stoichiometric changes with even single nucleobase additions or subtractions that may cause total bound RNA percentages to vary drastically, or specific lengths of RNA may elicit greater preference for other protein-protein or protein-RNA interactions. We aim to individually test these hypotheses in our future work. While the gel-shift analysis provides a general understanding of M2-1:RNA complex formation we cannot define a specific binding modality. Our image-based analysis is limited in its ability to capture the full possibilities of the M2-1:RNA complex formed, especially when considering the potential heterogeneity of the complex formed. The data collected here allows us to get an estimate for complex formation, which will need to be studied further in the future.

Our mutagenesis analysis provides insights into the specific residues implicated in RNA binding; a key cysteine residue at position 7 (C7) was identified as a key mediator for the interaction between M2-1 and short RNA sequences. Previously we have shown that C7 is a key residue of the CCCH motif due to its role in M2-1 to RNA binding (20). It has also been concluded previously that changes at C7 have decreased the binding affinity of M2-1 to viral RNA by 58%, using a time course colorimetric assay to quantitatively measure zinc binding (38). Changes in the zinc-binding motif will likely impact the oligomerization of M2-1, further inhibiting proper function. The importance of the C7 residue and the full impact on the formation of the M2-1 complex is a potential future direction of our preliminary analysis. The C7A mutant M2-1 eluted at the same position as *apo* M2-1 on SEC, but with a broader, trailing peak. This indicates that the tetrameric formation of M2-1 may still be possible and C7 is the crucial residue for the formation of higher-order oligomer complexes between M2-1 and longer RNA. The mutants of M2-1 utilized in this study were designed based on the structure of M2-1 in a complex with short SH7 RNA. Other potential residues were considered due to their changes in complex formation, including F9A, F23W, R9Y, and R151D. The only residue that led to a total abolishment of complex formation was C7 (Fig. 4), which was the focus of the study. However, this approach may have overlooked other potentially more crucial residues. We plan this analysis as a future direction of this work.

The investigation of the M2-1:RNA complex formation at various protein-to-RNA ratios revealed that local RNA concentration could modulate complex size, uniformity, and compactness. Interestingly, a prior morphological analysis of protein:RNA condensates formed by M2-1 with a 20-mer RNA derived from the SH gene-end RNA sequence depicted that condensate properties are modulated by the stoichiometric ratio of M2-1:RNA (37). Specifically, condensate size was modulated by RNA concentration, and saturating the RNA binding site of M2-1 caused a shift in preference from heterotypic protein:RNA interactions to self-homotypic interactions between the RNA binding site and other regions of M2-1, eventually resulting in the disappearance of the M2-1:RNA condensates (37). From our results, the higher oligomeric assembly of RNA to M2-1 and increased complex size with higher RNA concentrations indicate complicated dynamics highly sensitive to RNA abundance. These findings emphasize the importance of considering both protein and RNA concentrations in understanding complex formation. Other important considerations of complex formations are the post-translational modifications that M2-1 can potentially undergo. Phosphorylation of the RNA-M2-1 complex upregulates the binding of the M2-1 protein to viral RNA and will most likely play an important role in the complex formation. M2-1 must first be phosphorylated at the zinc-binding motif to enable binding to viral RNA during infection (38, 39). Phosphorylation affects the function of M2-1 binding *in vivo* but does not affect binding character *in vitro* (19), which is why it was not a focus of this study. This is a key component of understanding complex formation *in vivo* and should be further evaluated in future studies.

The stability analysis through RNase A digestion and EDTA treatment highlighted the robustness of the M2-1:RNA complex structure. The lack of significant structural changes in the complex particles when performing RNase A digestions and EDTA treatment following particle assembly suggests that interactions between the M2-1 tetramer and RNA may involve a burial of the zinc-binding domain of M2-1 along with sequestration of RNA, thereby serving an RNA-protective function. Alternatively, though we show that long RNA induces higher-order oligomerization, the RNA within the complex may be partially digested by the RNase A without structurally degrading the oligomer itself. Additionally, since the EDTA chelation assay was done following M2-1 and RNA co-incubation, we cannot rule out the possibility that zinc chelation before RNA binding may significantly affect complex structure and morphology. EDTA has been previously shown to outcompete zinc resulting in zinc stripping within a few minutes after the addition of EDTA (40). Additionally, some zinc-binding domains may not be refolded after the removal of EDTA and the addition of zinc. This emphasizes the conclusions stated on the robustness of the M2-1:RNA complex after the EDTA treatment.

The insights gained from this study have implications for our understanding of the biomolecular role of M2-1 in RNA regulation. The distinct binding behaviors with different RNA lengths and sequences indicate the potential for fine-tuned regulatory mechanisms. Future studies could delve deeper into the structural basis of these interactions. Our study employed RNA sequences shorter than 30 nucleotides, a limitation considering that cellular RNA and viral mRNA sequences *in vivo* are typically much longer. It is possible that these RNAs could interact with the M2-1 tetramer to form more complex and larger higher-order structures, a direction we plan to explore in future work.

In conclusion, the results presented in this study provide insights into the complex formation mechanisms, sequence preferences, binding characteristics, and interactions of M2-1 with different RNA lengths and sequences. Our findings may contribute initial steps toward elucidating the processes driving M2-1 colocalization with viral mRNAs into IBAGs, which may potentially stem from the cooperative manner in which RNA sequences of sufficient length bind to M2-1. Our findings underscore the complexity and specificity of interactions between M2-1 and RNA, which could aid further exploration into molecular mechanisms that govern the role of M2-1 role in RNA processing broadly and its potential implications in various host biological processes.

## MATERIALS AND METHODS

### Protein purification

The M2-1 construct was generated by inserting the M2-1 DNA from HRSV strain A2 into the vector pLICv1_His6-GFP-tev-yORF, allowing for the expression of M2-1 with a 6*His_GFP tag at its N-terminus. *Escherichia coli* BL21(DE3) was used as the host cell for M2-1 expression. The cells were cultivated at 37°C until the OD600 reached 0.8, and then the induction of expression was achieved by adding 0.5 mM IPTG. The cells were pelleted via centrifugation after an overnight incubation at 16°C. To purify the M2-1 protein, the pelleted cells were resuspended in high salt lysis buffer (50 mM sodium phosphate, pH 7.4; 500 mM NaCl; 5 mM Imidazole, pH 7.4; 10% Glycerol; 0.2% NP-40) and subjected to sonication for 15 minutes (3 seconds ON, 3 seconds OFF, 60% amplitude) on ice water. After centrifugation at 16,000 RPM for 40 minutes at 4°C, the soluble fraction containing the protein was collected and loaded onto a gravity cobalt column. Subsequent washing with high salt lysis buffer (50 mM sodium phosphate, pH 7.4; 1.5 M NaCl; 5 mM Imidazole, pH 7.4; 10% Glycerol) and elution with Elution buffer (50 mM sodium phosphate, pH 7.4; 500 mM NaCl; 250 mM Imidazole, pH 7.4; 10% Glycerol) yielded the purified M2-1 protein. The M2-1 protein was then subjected to TEV protease cleavage in a 20:1 wt/wt ratio overnight at room temperature in dialysis buffer (25 mM Tris-HCl, pH 8.0; 300 mM NaCl; 10% Glycerol; 1 mM DTT), resulting in efficient cleavage. Subsequent exchange into the buffer (50 mM MES pH 6.0; 150 mM NaCl; 5% Glycerol) and purification using a HiTrap Heparin HP column with a stepwise NaCl gradient further purified the M2-1 protein. The BioRad protein kit (BioRad Catalog # 1511901) was used for calibration of the Superdex 200 increase 10/300 column. The kit contains five peaks: the first peak is Thyroglobulin (670 KDa), the second peak is γ-globulin (158 kDa), the third peak is Ovalbumin (44 kDa), the fourth peak is Myoglobin (17 kDa), and the fifth peak is Vitamin B12 (1,350 Da).

### Electrophoretic mobility shift assay and analysis

RNA was incubated with increasing concentrations of M2-1 proteins in the binding buffer containing 20 mM HEPES pH7.5, 200 mM NaCl, 2 mM TCEP, and 5% glycerol. All the samples were incubated at room temperature for 1 hour and loaded on the 4% polyacrylamide native gel. The electrophoresis was pre-run at 100 V for about 30 minutes, and the samples were run for 1.5 hours. The gel was stained with the SYBR gold stain solution for 20 minutes. The visualization of RNA was detected in a ChemiDoc Imaging System. Each EMSA trial was performed in triplicate and representative images with clear backgrounds and minimal noise were used for image analysis. Scanned gel images were analyzed using the NIH program ImageJ (41) and with a protocol adapted from a label-free method of EMSA analysis for measuring dissociation constants of Protein-RNA Complexes (42). The images were first inverted, and the raw integrated density (RID) of each band was measured using ImageJ using uniform selection windows. When analyzing complexes formed by M2-1 with polyA RNAs of varying lengths, bands corresponding to free RNA were relatively sharp, and were thus used for density measurements, since in some cases there was either limited migration of the M2-1-RNA complex or the complex bands were spread out through the well. The only exception to this was for the M2-1 with poly9A gel, as the small RNA length made it difficult to stain and thus difficult to determine accurate density measurements, so the M2-1 and RNA complex bands were used instead. The results were then imported into spreadsheet software where a series of calculations were performed to extract binding percentages. The analysis was performed based on the free RNA bands seen on the gel. First, relative RID values were calculated by subtracting each RID value from the lowest RID value. Percentage RID values were subsequently calculated by dividing each relative RID by the largest relative RID and multiplying each value by 100. Finally, the percentage of binding/percentage of complex formation was calculated by subtracting each percentage RID value from 100. Binding percentage values were then imported into the nonlinear regression analysis and graphing software GraphPad Prism (version 9.00 for Mac; GraphPad Software, La Jolla CA; https://www.graphpad.com/). The software provided a suite of regression analyses, and the equations chosen for identifying curve fits were located under the tab “Nonlinear regression (curve fit)” within the “XY Analyses” category. Curve analysis was done with the total and nonspecific analysis. The specific equations used for regression analysis were located within the “Binding—Saturation” set of equations. Based on the goodness of the fit, which was surmised from the coefficient of determination R², three equations were selected for comparison: “One site—Specific binding,” “One site—Total and nonspecific binding,” and “Specific binding with Hill slope.” From these three equations, the equation with the best fit was utilized for graph generation. All equations provided dissociation constant (K_d_) values, and Hill coefficient values were determined from the “specific binding with Hill slope” equation for all gels. This coefficient was used in estimating levels of cooperativity involved in M2-1-RNA binding for each RNA length.

### Mutagenesis

The single-point mutations were introduced using the DpnI-dependent QuikChange site-directed mutagenesis method (Agilent), and the sequence of each construct was verified via DNA sequencing (Eurofins Genomics). All mutants were purified following the same procedure as the wild-type M2-1.

### RNase A treatment

RNase A treatment assays were used to test the stability of the M2-1:RNA complexes. Reactions were carried out with 10 ug of RNase A in the reaction buffer containing 20 mM HEPES, 200 mM NaCl, and 2 mM TCEP at room temperature for 0, 1, and 3 hour(s), respectively. Each reaction was stopped to make negative stain EM grids for imaging the complex particles.

### EDTA chelation assay

M2-1:RNA complexes were incubated at room temperature for 1 hour with the buffer 20 mM HEPES, 200 mM NaCl, and 2 mM TCEP. Next, the EDTA was mixed with the sample by the gradient final concentration as 0.5 µM, 2.5 µM, 5 µM, and 7.5 µM, separately. The EDTA chelation samples were gently mixed and incubated at room temperature for another 1 hour. Finally, the reaction samples were checked by 4% polyacrylamide native gel.

### Negative stain EM

Samples were diluted with the buffer 20 mM HEPES pH 7.5, 200 mM NaCl, 2 mM TCEP to the final concentration of 0.03 mg/mL and prepared on continuous carbon films supported by 400-mesh copper grids. A volume of 4 uL of the samples was absorbed into a freshly glow-discharged grid, washed with two drops of deionized water, and stained with two drops of freshly made 0.75% uranyl formate. Samples were imaged using a TSF Talos L120C electron microscope operating at 120 KeV and equipped with a TSF Ceta 4K × 4K charge-coupled device camera. Micrographs were collected at nominal magnifications of ×92,000 [1.563 Å/pix]. The micrographs were acquired at a defocus value of approximately −1.5 to −2.2 µm and electron doses of 25 e−/Å^2^. 2-D class averages of negative stain EM micrographs were processed using EMAN2.

## Data Availability

The data that support the findings of this study are available upon request from the corresponding author, B.L.

## SUPPLEMENTAL MATERIAL

Supplemental material jvi.01046-24-s0001.docx Fig. S1.

ASM does not own the copyrights to Supplemental Material that may be linked to, or accessed through, an article. The authors have granted ASM a non-exclusive, world-wide license to publish the Supplemental Material files. Please contact the corresponding author directly for reuse.
