# Supplementary material for: In vitro higher-order oligomeric assembly of the respiratory syncytial virus M2-1 protein with longer RNAs
Source: J Virol. 2024 Jul 17;98(8):e01046-24. doi: 10.1128/jvi.01046-24 (PMC11334520; doi:10.1128/jvi.01046-24)
Supplement: Supplemental material — Fig. S1. [file jvi.01046-24-s0001.docx]

**Supplemental Figures**

**
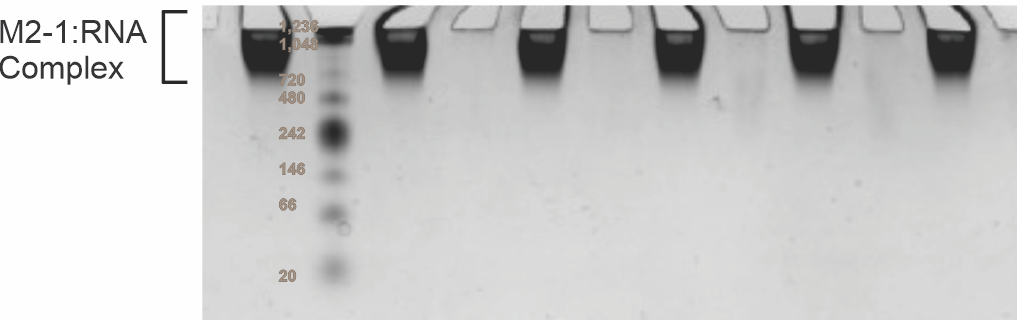
**

**Supplemental Fig. 1.** Coomassie blue stained SDS-PAGE gel showing the size of the M2-1:RNA complex after treatment with RNase A at the 1, 2-, 3-, 4-, and 5-hour timepoints, respectively. This gel was previously stained with SYBR-Gold and shown in Fig. 6 of the main text.
